# Health-related locus of control and health behaviour among university students in North Rhine Westphalia, Germany

**DOI:** 10.1186/1756-0500-5-703

**Published:** 2012-12-29

**Authors:** Stefanie M Helmer, Alexander Krämer, Rafael T Mikolajczyk

**Affiliations:** 1 Bremen Institute for Epidemiology and Prevention Research, University of Bremen, Bremen, Germany; 2 School of Public Health, Bielefeld University, Bielefeld, Germany; 3 Department of Epidemiology, Helmholtz Centre for Infection Research, Braunschweig, Germany; 4 Hannover Medical School, Hannover, Germany

**Keywords:** Locus of control, Health behaviour, Students, Cross-National Student Health Study

## Abstract

**Background:**

Health control beliefs were postulated to be associated with health behaviour. However, the results of studies assessing these associations suggest that they might not be universal. Among young adults associations have been reported, but the evidence is limited. The objective of this analysis was to re-examine these associations in a sample of university students in Germany.

**Findings:**

Data from a multicentre cross-sectional study among university students in North Rhine-Westphalia, Germany was used (N=3,306). The Multidimensional Health Locus of Control Scale with three dimensions (one internal and two external) and six aspects of health behaviour (smoking habits, alcohol use, drug consumption, being over-/ or underweight, physical activity, and importance of healthy nutrition) were evaluated. Students with stronger internal locus of control paid more attention to healthy nutrition and displayed a higher level of physical activity. Individuals with a stronger belief in health professionals were less likely to use drugs and paid more attention to healthy nutrition. Furthermore, higher scores in the second external locus of control dimension (beliefs in luck or chance) were associated with a higher likelihood of current smoking, lower physical activity and less attention to healthy nutrition.

**Conclusions:**

Students engaged more strongly in unhealthy behaviour if they believed that luck determines health. In contrast, believing in having control over one’s own health was associated with more healthy behaviour. These findings support the need to consider health control beliefs while designing preventive strategies in this specific population.

## Findings

### Health control beliefs

Having control over one’s health has been postulated to have effects on personal health behaviour [1]. Furthermore, Shapiro et al. proposed that having control is not as important as the belief of having control [2]. This subjective understanding of control beliefs regarding health can be conceptualised in different ways [3]. The most common approach is the Health Locus of Control (HLOC) construct, which links the individual health behaviour to external or internal factors [4]. The corresponding Multidimensional Health Locus of Control Scale (MHLC) is based on three dimensions: 1. an Internal Locus of Control (ILOC) that considers health as a product of personal decisions and a healthy or unhealthy lifestyle, 2. an External Locus of Control, subdivided into two dimensions: control by chance (Chance Locus of Control, CLOC) (e.g. luck determines health), and 3. control by “powerful others” (Powerful Others Locus of Control, PLOC) (e.g. following advice by authorities determines health) [1,5].

The HLOC theory suggests that high ILOC is linked to healthy behaviour. In contrast, beliefs in luck (or more general: chance) should increase likelihood of negative forms of health behaviour, because health is seen as independent of personal health behaviour [1,6-8]. The control by “powerful others” can be related to a good compliance, but requires directives by the “authority” and its connection to health behaviour is more ambiguous [9].

Empirical studies assessing the links proposed by the HLOC theory are inconsistent in their findings. In general, studies based on larger samples found expected associations [9-12], whereas studies using smaller samples did not [13,14]. Furthermore, within the LOC dimensions, findings differed by form of health behaviour, with clearer associations for smoking [10,11,15,16] or less physical activity [11,17] than for alcohol consumption [9,11,13] or unhealthy nutrition [18]. Several explanations were proposed for this complex picture [11,13,19]. First, given the rather weak associations, it is possible that smaller studies did not have sufficient power to detect them. Nevertheless, weak associations which affect the whole population can still be of substantial public health relevance [19]. On the other hand, larger studies typically had heterogeneous samples; therefore the observed strength of associations might have been attenuated. Second, within the theory of planned behaviour Ajzen proposed that in order to consider a given health behaviour an individual’s responsibility, the knowledge of the implications of this health behaviour is necessary [20]. However, the knowledge of health implications is not universal and cannot be assumed a priori. For instance, about half of the population in a survey in Wales did not consider nutrition to be a relevant factor for their long term health [21]. While the above findings were published in 1986, it is likely that lack of health behaviour related knowledge remains a problem. The assumption that lack of knowledge can provide an explanation as to why health control beliefs do not affect behaviour, is supported by the findings from analyses stratifying study samples by educational status and demonstrating stronger associations among those with better education [11]. Third, Hunt & Martin suggested that health behaviour can be strongly influenced by habits [22]. However, differences can not only exist across forms of health behaviour with respect to the degree by which they are determined by habits, but also across age groups with regard to how firmly behavioural habits are established

Given these considerations, university students could be a population in which the associations between HLOC and health behaviour and therefore may be particularly interesting for assessing it. University students are in an especially interesting biographic state to newly determine their health behaviour and might experience more freedom in making personal choices about their health behaviour than earlier and later in life [18,23,24]. In contrast to school-age children, most university students are not dependent on their parents and at the same time do not have to assume the responsibility for their own families. In this period of life, students and young people can explore different life directions and explore own life style choices [23]. Furthermore, some forms of unhealthy behaviour such as risky alcohol consumption and illicit drug consumption peak in this age group [24,25].

Restricting the study population to university students has the advantage of reducing heterogeneity and focusing on healthy, educated persons who are likely to be most aware of the implications of health behaviour [26,27]. Understanding health behaviour of university students could also open important venues of intervention. For example, health behaviour of students that is linked to “powerful others” health beliefs (PLOC) might be of interest for health campaigns. Only three of the previous studies assessed locus of control among university students [14,18,19] and only one study examined the association between health beliefs and health behaviour among this specific group [19]. The latter study included students from several European countries and therefore there was a higher heterogeneity in the sample, which possibly affected the findings. Therefore, the purpose of this study was to assess associations between the dimensions of HLOC and health behaviour in a homogenous sample of university students from one country. Germany was chosen by way of convenience, and similar studies are being prepared for other countries in the network of Cross-National Student Health Study [26]. It was hypothesized that students with high internal health beliefs will display more healthy behaviour, whereas those with high chance locus of control beliefs will show more unhealthy behaviour. Furthermore, we assumed that high scores in the powerful others dimension will be associated with a higher likelihood of health behaviour in areas that are often topics of health campaigns (i.e. smoking, high alcohol consumption).

### Methods of the study

#### Sample

The dataset used in this analysis originates from a study of health and health behaviour among university students in the most populated federal state of Germany, North Rhine-Westphalia (NRW) [28,29]. This multicentre cross-sectional study was conducted in 2006/2007. Students from 12 (out of 13) universities in NRW and 4 universities of applied sciences (selected randomly out of 25 in NRW) were invited to complete a self-administered questionnaire. This ratio between universities and universities of applied sciences was chosen to match the ratio of 3:1 of students from general and applied universities. The study sample was representative of students in NRW with respect to study duration, nationality and migration background. The proportion of female participants in the study sample was slightly higher compared to females among all students in NRW. Courses were selected partly randomly and partly by convenience, and students were invited to fill out the self-administered questionnaire at the end of a lecture. Initially, a balanced sample of students of natural sciences and social sciences was planned. Due to organisational difficulties and variation in response rates this plan was not fully realised. In the final sample, students of medicine and health sciences, educational sciences as well as sport sciences were overrepresented [29].

Participation in the study was voluntary and anonymous. Students were informed that by completing the questionnaire they were providing their informed consent to participate. They were also informed that they could terminate participation at any point while filling out the questionnaire. The permission to conduct the study was granted by the participating institutions which are listed in the acknowledgments. The response rate varied across the universities between 69.3% and 100% (average 87.9%). The final sample included 3,306 students.

#### Measures

##### Health locus of control

Health locus of control was assessed by the German translation of the Multidimensional Health Locus of Control Scale (MHLC) developed by Wallston [24,30]. This scale distinguishes between the “internal” (ILOC), and two external (“powerful others” – PLOC and “chance” - CLOC) dimensions. ILOC refers to the extent that one’s behaviour is responsible for the health or illness of an individual. PLOC describes the ascribed influence by healthcare professionals for personal health and CLOC encompasses the degree to which health or illness depends on chance. The MHLC uses five-point Likert scales measuring the extent to which subjects agree or disagree with the statements (1=strongly disagree; 5=strongly agree). Each of the three dimensions was assessed by three statements regarding the relevant health beliefs (Table 1). For each dimension the sum of items was calculated. A high score indicated a high importance of the locus of control in the given dimension, i.e. the higher the ILOC-score, the higher the importance of ILOC.

**Table 1:** Health locus of control ratings by sex

		**Female**		**Male**	
**Health locus of control dimensions**	**Items**	**N**	**Mean (SD)**	**N**	**Mean (SD)**
Internal (ILOC)	* The main thing which affects my health is what I myself do.	1688	11.21 (1.86)	1498	11.65 (1.80)
	* I am in control of my health.				
	* If I get sick, it is my own behaviour which determines how soon I get well again.				
Powerful others (PLOC)	* Regarding my health, I can only do what my doctor tells me to do.	1677	6.98 (1.70)	1502	7.14 (1.89)
	* Having regular contact with my physician is the best way for me to avoid illness.				
	* Health professionals control my health.				
Chance (CLOC)	* My good health is largely a matter of good fortune.	1677	7.07 (1.77)	1504	7.24 (1.92)
	* Luck plays a big part in determining how soon I will recover from an illness.				
	* If it’s meant to be, I will stay healthy.				

SD – standard deviation.

#### Health behaviour

Six different forms of health behaviour were included in the analysis:

• Smokers were defined as individuals who reported cigarette smoking in the last six months. Participants who were not current smokers but reported smoking at some point during their life were categorised as former smokers. Students who never smoked were defined as non-smokers.

• Alcohol consumption was measured as frequency of drinking in the last three months using the following categories: at least every day, several times per week, once a week, less than once a week, never.

• Drug intake in the last three months was investigated for marijuana, cocaine, amphetamines, and antipsychotic drugs. Consumption of one or more drugs in the last three months was coded as ‘yes’ for drug intake. We used only two categories, since less than one per cent of the study population consumed more than one drug in the past three months.

• Being over/underweight was defined using Body-Mass-Index (BMI). BMI was calculated using self-reported weight in kilograms divided by height in meters squared (kg/m^2^). Following the standard proposed by the World Health Organisation [31], we used three categories for classifying weight; normal weight was defined as a BMI between 18.5 to 25; underweight was defined as a BMI below 18.5 and overweight as a BMI above 25.

• Physical activity was assessed by asking participants the following question: How many times do you engage in a physical activity (e.g. sports, physical work) in a normal week that takes at least 20 minutes and makes you breathe harder than normal? Possible responses were: less than once per week, 1 to 2 times per week, at least 3 times per week.

• Behaviour with respect to healthy nutrition was measured by the self-reported importance of eating healthy foods. The scale is a 5-point Likert scale with the following answers: very important, somewhat important, important, somewhat unimportant and very unimportant.

#### Statistical analysis

Descriptive analysis was performed using tabulations by sex. In the next step, multinomial (alcohol consumption, smoking habit, BMI and importance of healthy nutrition) and binary (drug intake) logistic regression were used to assess independent associations between health locus of control and health behaviour. All models were composed of sex and age, and the three HLOC scales as independent variables. For these analyses, the three HLOC scales and age were used as continuous variables and the reported odds ratios correspond to one unit change in dimensions score. Before entering the independent variables in the model, the multicollinearity between independent variables was assessed based on tolerance and VIF coefficients. Standard threshold values (tolerance >0.01) were applied [32]. Since only 1% to 9% of the variance of the independent variables were explained by the other variables, no multicollinearity problems were diagnosed. Data analysis was performed using the statistical program SPSS for windows, version 17.0.

## Results

### Description of the sample

More than half of the student sample (52%) were females; the mean age was 23 years (SD = 2.2). 87% of the students were born in Germany. Among the three HLOC scales, ILOC scored the highest with an average of 11.43 (the highest possible value for all scales was 15). The mean values of PLOC and CLOC were similar (7.07/7.15). The scores for HLOC for all three scales differed by sex, with higher scores on average in males than in females. The results are presented in Table 1.

### Health behaviour

In total, 22.1% of the students were current smokers and 32.5% reported a regular intake (at least several times per week) of alcohol. About 10% reported consumption of drugs like marijuana, cocaine, amphetamines, and antipsychotic drugs in the last three months. In 16.8% of the sample, the BMI was over 25 kg/m^2^ and in 4.5% under 18.5. Over 40% of the students reported to be physically active less than once a week and 8.1% reported paying little attention to healthy nutrition (Table 2).

**Table 2:** Health behaviour in the study sample by sex

		**Female N=1699**		**Male N=1522**		
**Health behaviour**		**N**	**%**	**N**	**%**	**p-value**
Smoking	Non-smoker	1081	63.6	918	60.3	0.145
	Ex-smoker	219	12.9	228	15.0	
	Smoker	378	22.2	341	22.4	
	Missing	21	1.2	35	2.3	
Alcohol	Never	16	0.9	65	4.3	<0.001
	Less than once a week	673	39.6	305	20.0	
	Once a week	470	27.7	394	25.9	
	Several times every week	334	19.7	638	41.9	
	At least every day	16	0.9	65	4.3	
	Missing	7	0.4	11	0.7	
Drug consumption	No drug intake	1539	90.6	1275	83.8	<0.001
	Drug intake	117	6.9	198	13.0	
	Missing	43	2.5	49	3.2	
BMI	Underweight	129	7.6	18	1.2	<0.001
	Normal weight	1305	76.8	1137	74.7	
	Overweight	205	12.1	352	23.1	
	Missing	60	3.5	15	1.0	
Physical activity	at least 3 times a week	539	31.7	780	51.2	<0.001
	1 to 2 times a week	691	40.7	463	30.4	
	Less than once a week	461	27.1	263	17.3	
	Missing	8	0.5	16	1.1	
Importance of healthy nutrition	Very important	429	25.3	292	19.2	<0.001
	Somewhat important	825	48.6	596	39.2	
	Important	374	22.0	432	28.4	
	Somewhat unimportant	59	3.5	142	9.3	
	Very unimportant	10	0.6	52	3.4	
	Missing	2	0.1	8	0.5	

For five of the six aspects of health behaviour there was a difference by sex, with women reporting more healthy behaviour with respect to alcohol, drug use, and nutrition (data not shown). In contrast, women reported lower physical activity than men. In terms of BMI, female students were more likely to be underweight and less likely to be overweight. Current smoking and overweight were more common among older students. In contrast, older students were more likely to pay attention to healthy nutrition.

### Associations between health behaviour and the dimensions of HLOC

Results of the regression analyses are presented in Table 3. Higher ILOC was associated with a higher physical activity and a higher importance of healthy nutrition. Higher PLOC was associated with a lower drug use, not paying attention to healthy nutrition and less physical activity during a typical week. Higher ratings in the CLOC dimension were associated with a higher likelihood of being a current smoker, drinking alcohol at least once a day, less physical activity and less attention to healthy nutrition. In terms of explained variation in health behaviour the models displayed rather low values (Nagelkerke’s-R^2^ below 0.1 for most models, apart from the regression models for alcohol consumption as well as the importance of healthy nutrition with the HLOC scales and socio-demographic variables which had a Nagelkerke’s-R^2^ of 0.11), the odds ratios however, indicated strong effects for selected groups. For example, per unit change in CLOC dimension score, the probability of being a current smoker (in comparison to never-smoker) increased by 9%. For the importance of healthy nutrition the probability of “very unimportant” (in comparison to “very important”) decreased by 36% per one unit of ILOC score.

**Table 3:** Associations between three dimensions of the Multidimensional Health Locus of Control Scales and different forms of health behaviour adjusted for age and sex* (results from binary logistic and multinomial regression indicate odds ratio per unit change in HLOC dimension score, separate models for each form of behaviour**)

		**ILOC**	**p-value**	**PLOC**	**p-value**	**CLOC**	**p-value**
**Behaviour**		**OR (95% CI)**		**OR (95% CI)**		**OR (95% CI)**	
Cigarette smoking (N=3229 )	Non-Smoker	1		1		1	
	Ex-Smoker	1.01 (0.95-1.07)	0.826	0.95 (0.89-1.01)	0.084	0.96 (0.91-1.02)	0.230
	Smoker	1.01 (0.96-1.06)	0.778	0.97 (0.92-1.02)	0.165	1.09 (1.04-1-15)	0.001
Alcohol consumption (N=3271)	Never	1		1		1	
	Less than once a week	1.03 (0.96-1.11)	0.449	1.03 (0.95-1.11)	0.494	0.97 (0.90-1.04)	0.397
	Once a week	1.04 (0.97-1-12)	0.318	0.99 (0.92-1.07)	0.847	0.96 (0.89-1.04)	0.318
	Several times every week	1.04 (0.97-1-12)	0.297	0.94 (0.87-1.01)	0.111	1.02 (0.95-1.10)	0.563
	Every day or more	1.07 (0.93-1.23)	0.363	0.95 (0.82-1.09)	0.442	1.19 (0.97-1.07)	0.013
Drug intake (N=3189)	No	1	0.801	1	0.012	1	0.319
	Yes	0.99 (0.93-1.06)		0.92 (0.85-0.98)		1.04 (0.97-1.11)	
Weight (N=3154)	Underweight	0.93 (0.84-1.02)	0.116	1.10 (0.99-1.22)	0.069	0.91 (0.82-1.01)	0.068
	Normal weight	1		1		1	
	Overweight	0.98 (0.93-1.04)	0.562	1.04 (0.99-1.10)	0.133	1.04 (0.98-1.09)	0.019
Physical activity (N=3263)	At least 3 times a week	1		1		1	
	1 to 2 times a week	0.87 (0.83-0.91)	<0.001	1.08 (1.03-1.13)	0.002	1.06 (1.01-1.11)	0.019
	Less than once a week	0.86 (0.81-0.91)	<0.001	1.07 (1.01-1.13)	0.026	1.12 (1.06-1.18)	<0.001
Importance of healthy nutrition (N=3281)	Very important	1		1		1	
	Somewhat important	0.89 (0.84-0.94)	<0.001	1.02 (0.97-1.08)	0.458	1.04 (0.99-1.10)	0.140
	Important	0.79 (0.74-0.84)	<0.001	0.96 (0.90-1.02)	0.135	1.10 (1.04-1.17)	0.001
	Somewhat unimportant	0.79 (0.72-0.87)	<0.001	0.90 (0.82-0.99)	0.025	1.25 (1.14-1.37)	<0.001
	Very unimportant	0.64 (0.55-0.74)	<0.001	0.83 (0.71-0.97)	0.016	1.29 (1.11-1.49)	0.001

* The first category of each health behaviour variable in the table indicated the reference group.

ILOC – internal locus of control.

PLOC – external locus of control dimension “powerful others”.

CLOC – external locus of control dimension “chance”.

OR – odds ratio.

CI – confidence interval.

** All models were composed of sex and age, and the three HLOC scales as independent variables.

## Discussion

In this multicentre survey of students in Germany, the associations between health beliefs and health behaviour were assessed. Among the three studied HLOC dimensions, ILOC had the highest scores, whereas the scores for PLOC and CLOC were nearly equal and much lower. Students reported a frequent consumption of alcohol, relatively frequent physical activity, but a low importance of healthy nutrition. Multivariable analyses showed multiple associations between the HLOC dimensions and selected health behaviour forms: However, these associations differed across the dimensions indicating an importance of a holistic view on health control beliefs.

In general, HLOC scales only accounted for a small fraction of variation in health behaviour, which is in agreement with previous studies in this field [11,19]. However, this finding led to different interpretations in previous research. On the one side, Calnan [11] raised doubts about the importance of the HLOC construct and suggested that health behaviour may not be associated with beliefs regarding control of health but rather with concerns over risky health behaviours like smoking or alcohol consumption. On the other side, Steptoe and Wardle [19] took a different position and argued that the effects of health control beliefs were rather strong in population terms in contrast to other psychological factors like social support. They also pointed out that correlation measures (and therefore their derivate “explained variance” in linear model or its equivalent in logistic regression) do not convey the public health importance of an association. Explained variation is driven by the effects observed in the vast majority of a population, but still there can be specific characteristics strongly related to specific risk behaviours (for example people with very high scores on PLOC have substantially higher likelihood of being current smokers). Identifying such subgroups can help in the development of targeted interventions [19].

In contrast to some previous studies among working age [10,11] or adolescent samples [15,16], there were fewer associations between the examined forms of health behaviour and ILOC. ILOC was neither associated with a higher likelihood of being a former or current smoker, nor for more frequent drinking, nor for using illicit drugs. However, high ratings on the ILOC scale were associated with an increased chance of more physical activity during a normal week and with paying more attention to healthy nutrition. This is consistent with the theory postulated by Steptoe and Wardle [19] that ILOC has stronger effects on health maintenance behaviour (e.g. healthy nutrition) [33] than on multiply determined risk behaviours (e.g. smoking, alcohol consumption and drug use).

In our study, ILOC was not related to a higher likelihood of being under- or overweight. Adolfsson et al. [34] reported significant associations between the two but they focussed on participants in a weight losing programme. In this subgroup, the association with ILOC may be due to the fact that losing weight is to some extent associated with goal attainment. In general, the observed lack of association can be linked to the fact that weight is not a direct outcome of a single aspect of behaviour and therefore the association between health beliefs and weight can be more complex.

For PLOC, our findings with regards to alcohol consumption were in line with data from previous studies among students as well as adults [11,19]. Additionally, our findings indicated an association between PLOC and drug intake. To our knowledge, no other study has evaluated this association. Whereas a positive correlation between PLOC and smoking was found in a population-based study by Bennett et al. [12], no association was observed in our study. It could be argued that behaviour with short-term consequences is more affected by PLOC than behaviour with long-term consequences. Alcohol and drug use can be associated with immediate health complaints like injuries, memory loss or sexual harassment that are directly visible for students [33]. In contrast, students might not feel to be at risk of health complaints due to smoking because smoking does not affect their immediate state of health and related diseases appear in a distant future [35]. The observation that students with higher CLOC beliefs have higher odds to be current smokers is in agreement with a population-based study in Wales [10]. Frequent alcohol consumption was associated with higher CLOC scores in our study. These results agree with the previous study addressing HLOC in university students [19]. Finally, our results regarding the importance of healthy nutrition were in line with another population-based study in Wales [12]. In summary, CLOC demonstrated reversed associations to ILOC as expected in the HLOC theory. Interestingly, CLOC was the only HLOC dimension associated with smoking. Given the omnipresent smoking prevention advertisement and increased awareness of consequences of smoking, being a smoker has to be associated with beliefs in chance and vice versa smoking prevention based on fear appeal might not be effective for those with strong beliefs that chance determines their health.

### Strengths and limitations

The strength of the present study was the large homogeneous sample of students from multiple institutions, but there were also several limitations. The MHLC scale was applied in a reduced form with only three items for each dimension, which was less than in other studies (e.g. [36]); this may have affected the precision of the measurement. Despite the self-administered questionnaire, there could have been some reporting bias for health behaviour. For healthy nutrition only the importance, but not the actual behaviour was measured and these two concepts may not be equivalent. Additionally, the measurement of smoking habits did not allow a distinction between occasional and regular smokers. Additionally, we included BMI as a measure of health with respect to weight which does not take a person's body fat content into account.

Another limitation was the lack of measurement of the value of health in our survey. Several previous studies concluded that the value of health is a moderator variable with respect to HLOC and health behaviour [6,10,12,37]. However, there are studies which do not confirm this moderator effect [19].

Overall, the sample of the current survey (as a regional sample) is not representative for all students in Germany. Furthermore, the sample differed from the total population of students in NRW in terms of study subjects and gender distribution. This may have had an impact on reported distributions of forms of health behaviour and potentially also on the strength of observed associations. Finally, the cross-sectional study design does not allow for causal inferences.

## Conclusions

Associations between HLOC dimensions and health behaviour have been shown among university students in Germany. ILOC was associated with health maintenance behaviour. CLOC had impact on more aspects of health behaviour than ILOC. As expected, both dimensions of the external locus of control produced contrasting findings: higher ratings for PLOC were associated with lower risk behaviour, whereas higher ratings for CLOC with higher risk behaviour. These findings support the need to consider distinct dimensions of health control beliefs while designing preventive strategies among students.

## Competing interests

The study was supported by the German statutory health insurance company Techniker Krankenkasse and by the statutory accident insurance institution of NRW Unfallkasse North Rhine-Westphalia, former Landesunfallkasse North Rhine-Westphalia. Both institutions were co-sponsoring the study. None of the sponsors took any role in the interpretation of the data, writing the manuscript and the decision-making about publishing.

## Authors’ contributions

SH planned and carried out the analyses, and wrote the manuscript. AK contributed to the design of the study and guided the data collection. RM participated in designing and coordinating the study as well as in performing the statistical analyses and writing and revising the paper. All authors read and approved the final manuscript.
